# Supplementary material for: A reference haplotype panel for genome-wide imputation of short tandem repeats
Source: Nat Commun. 2018 Oct 23;9:4397. doi: 10.1038/s41467-018-06694-0 (PMC6199332; doi:10.1038/s41467-018-06694-0)
Supplement: Supplementary file 1 — Supplementary Information [file 41467_2018_6694_MOESM1_ESM.pdf]

Supplementary Information for

A reference haplotype panel for genome-wide imputation of short tandem repeats

Saini *et al.*

Supplementary Figure 1

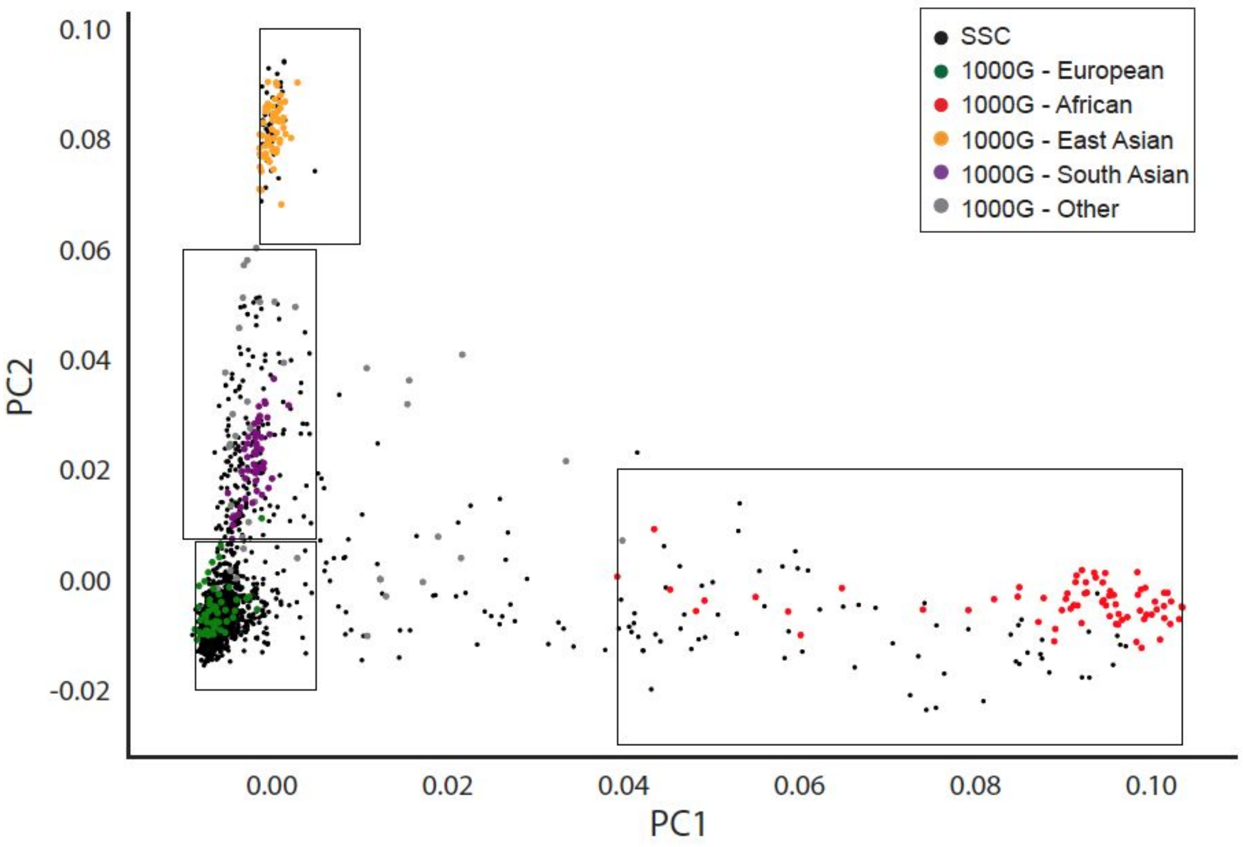

Analysis of SSC populations. Principal component analysis was performed using SNP genotypes from the SSC and 1000G cohorts. Boxes show inferred ancestry groups based on 1000 Genomes samples. Boxes for European, East Asian, South Asian, and African populations contain 1,585, 39, 172, and 69 SSC samples respectively. 51 SSC samples could not be confidently assigned to a population group.

Supplementary Figure 2

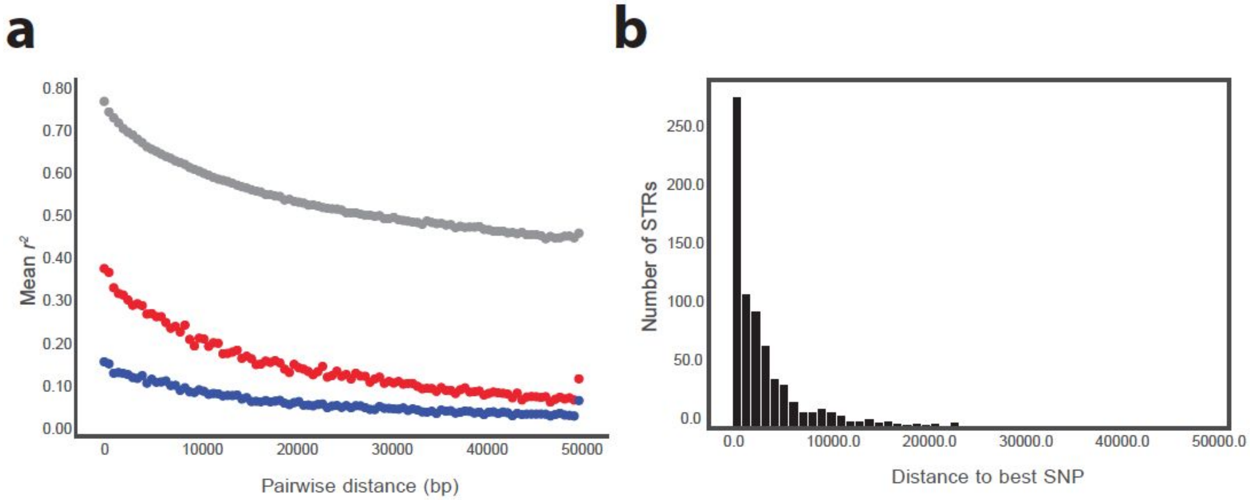

a. SNP-SNP LD is stronger than STR-SNP LD. Gray dots give the average pairwise SNP-SNP LD as a function of distance. Red dots give length r^2 computed as the squared Pearson correlation between STR length and SNP genotype (0, 1, 2). Blue dots give the allele r^2, defined as the squared Pearson correlation between each SNP and each STR allele treated as a separate bi-allelic marker. **b. Distribution of distances from each STR to its best tag SNP.** The best tag SNP is defined as the SNP within 50kb with the highest length r^2. The x-axis gives distance in bp.

Supplementary Figure 3

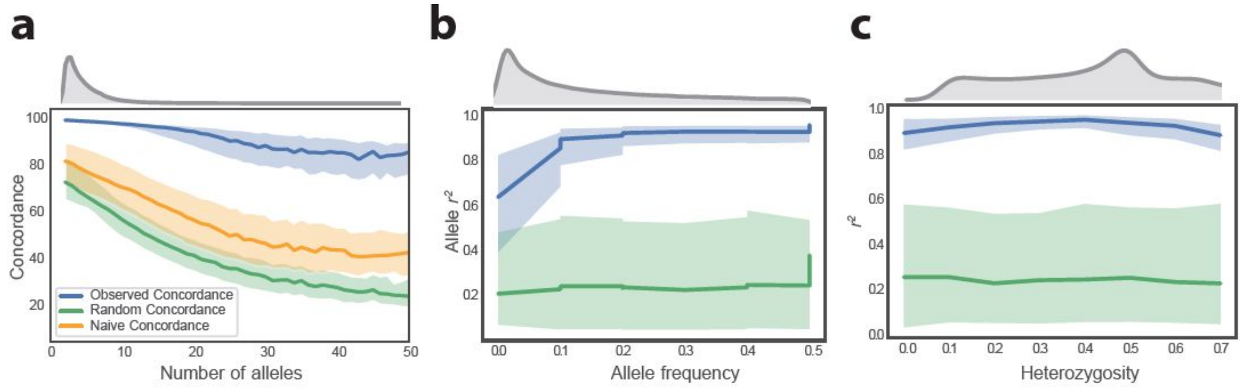

Imputation performance is strongest at the least polymorphic STRs. Plots show per-locus concordance vs. the number of alleles (**a**), allelic r^2 vs. allele frequency (**b**), and length r^2 vs. heterozygosity (**c**). Upper gray plots give the relative frequency of points along the x-axis. Blue denotes observed per-locus values, green denotes values expected under a random model and orange denotes values expected under a naive model as defined in the **Online Methods**. Solid lines give median values for each bin and filled areas span the 25th to 75th percentile of values in each bin. X-axis values for **a. b.,** and **c.** were binned by 1, 0.05, and 0.1, respectively.

Supplementary Figure 4

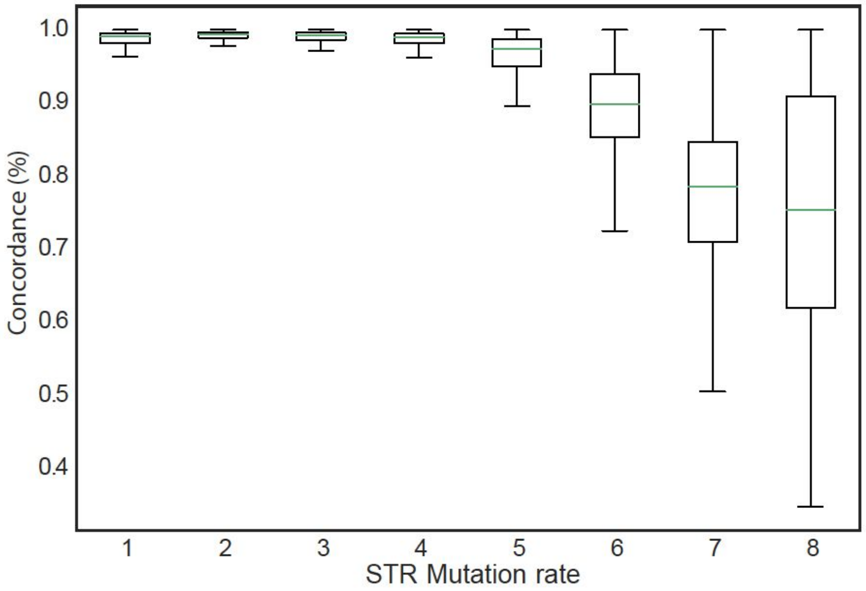

Imputation concordance vs. mutation rate. The x-axis gives the estimated mutation rate of each locus and y-axis gives concordance between imputed vs. HipSTR genotypes at each locus based on the leave-one-out analysis in SSC samples. Mutation rates were inferred by correlating local sequence heterozygosity with observed population-wide STR variation using the method described in Gymrek, *et al*[1]. Green lines give median values. Boxes span from the 25th percentile (Q1) to the 75th percentile (Q3). Whiskers extend to Q1-1.5*IQR (bottom) and Q3+1.5*IQR, where IQR gives the interquartile range (Q3-Q1).

Supplementary Figure 5

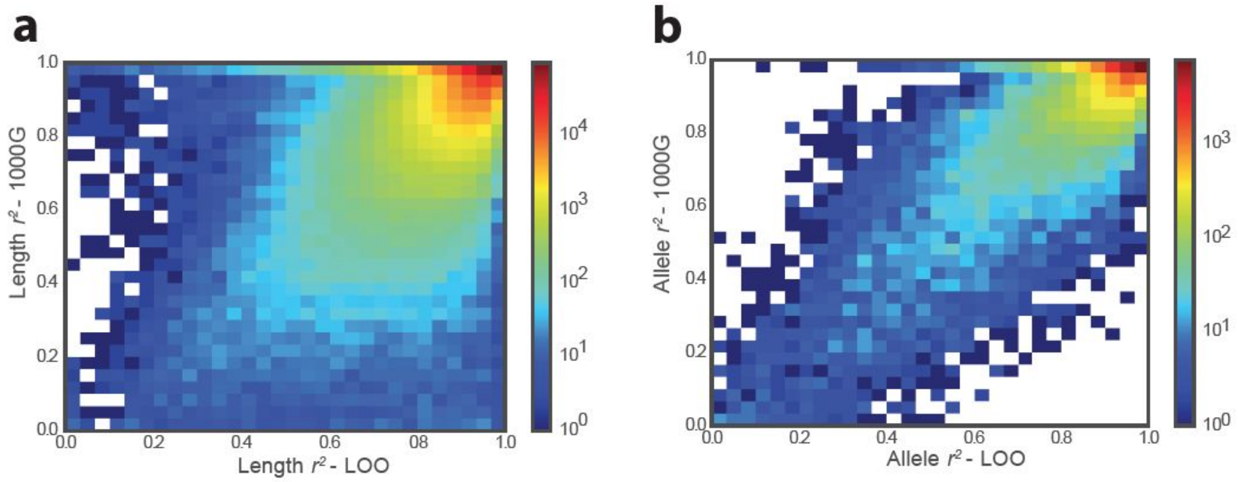

Comparison of per-STR imputation metrics in the SSC dataset (leave-one-out analysis) vs. in the 1000 Genomes European samples. a. and **b.** compare per-locus length r^2 and allelic r^2, respectively. Color scale gives the $\log_{10}$ number of STRs represented in each bin. 1000 Genomes values are based on comparing HipSTR genotypes obtained from deep WGS for 49 European samples vs. STR genotypes imputed into 1000 Genomes Phase 3 SNP data obtained from low coverage WGS.

Supplementary Figure 6

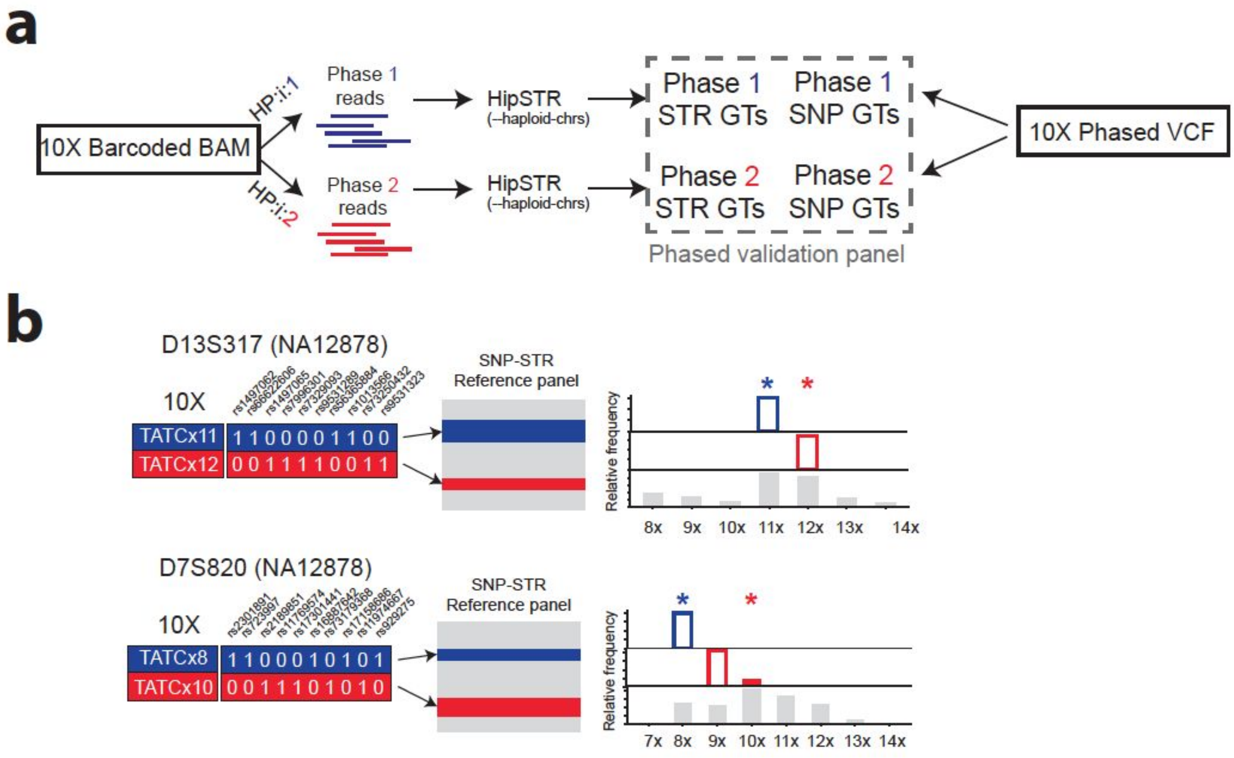

Evaluating imputation and phasing accuracy using 10X Genomics. a. Schematic of pipeline to create a phased SNP-STR validation set in NA12878. Barcoded BAMs were separated by phase and HipSTR was called in haploid mode separately on each set of reads. HipSTR genotypes from each read set were concatenated to form phased diploid genotypes. Phased STR and SNP genotypes were combined into a single phased validation panel. **b. Imputation vs. 10X results at example STRs.** Representative SNP-STR haplotypes are shown for NA12878 at two CODIS STRs. Blue denotes “phase 1” and red denotes “phase 2” as annotated in the 10X data. Values for each SNP (denoted by rsids) are 0 for the reference allele and 1 for the alternate allele. “10X” on the left denotes phased STR genotypes obtained using the pipeline in **a.** In each example all SNPs shown were identically genotyped in the 1000 Genomes Project panel and by 10X. Histograms on the right indicate STR allele frequencies in the SSC reference panel for the phase 1 SNP haplotype (blue), phase 2 SNP haplotype (red), and across the entire panel (gray). Filled bars give the imputed STR allele for each allele and stars give the expected value based on 10X genotypes. Both alleles at the top locus (D13S317) were imputed correctly. The second allele at the bottom locus (D7S820) was imputed incorrectly, likely because most haplotypes matching NA12878 contain 9, rather than 10, copies of TATC.

Supplementary Figure 7

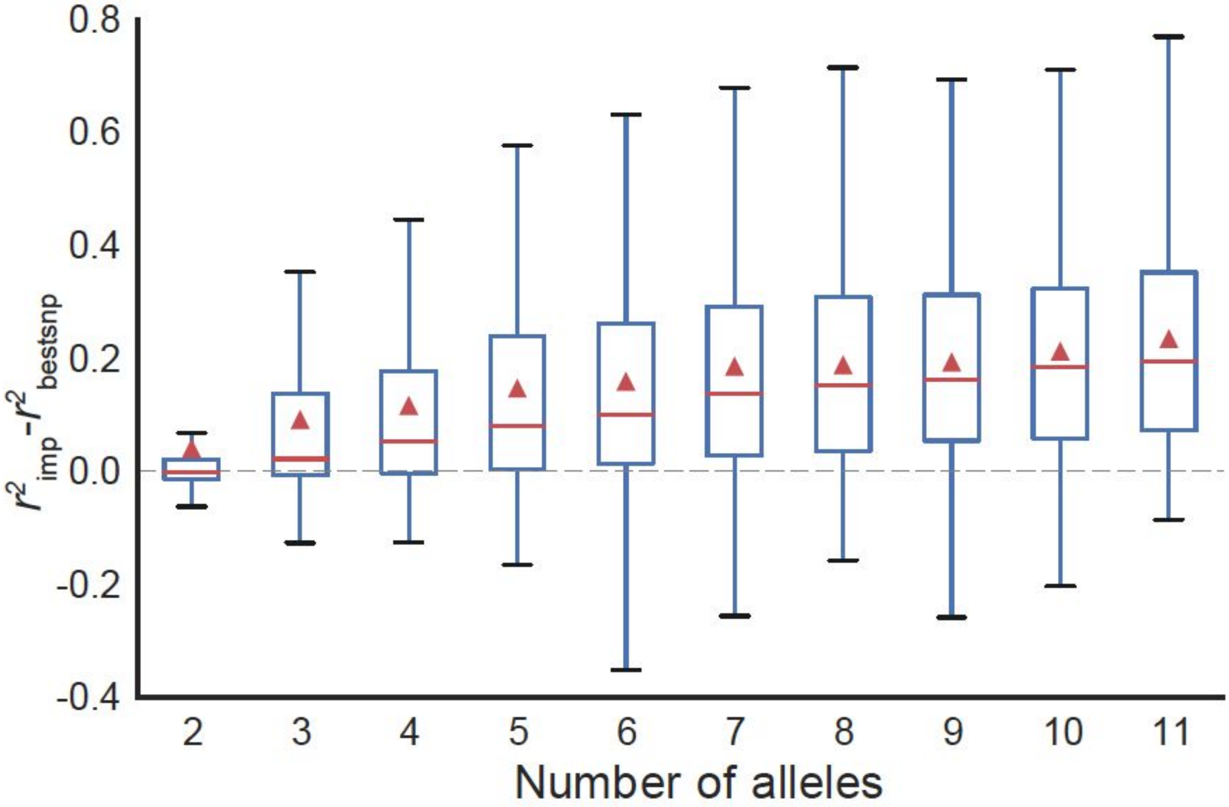

Gain in length r^2 for imputed STR genotypes compared to the best tag SNP vs. number of STR alleles. Data is shown for chr21 only. Red lines give medians and red triangles give mean values. Boxes span from the 25th percentile (Q1) to the 75th percentile (Q3). Whiskers extend to Q1-1.5*IQR (bottom) and Q3+1.5*IQR, where IQR gives the interquartile range (Q3-Q1). The best tag SNP is defined as the SNP within 50kb of the STR with the highest length r^2.

Supplementary Figure 8

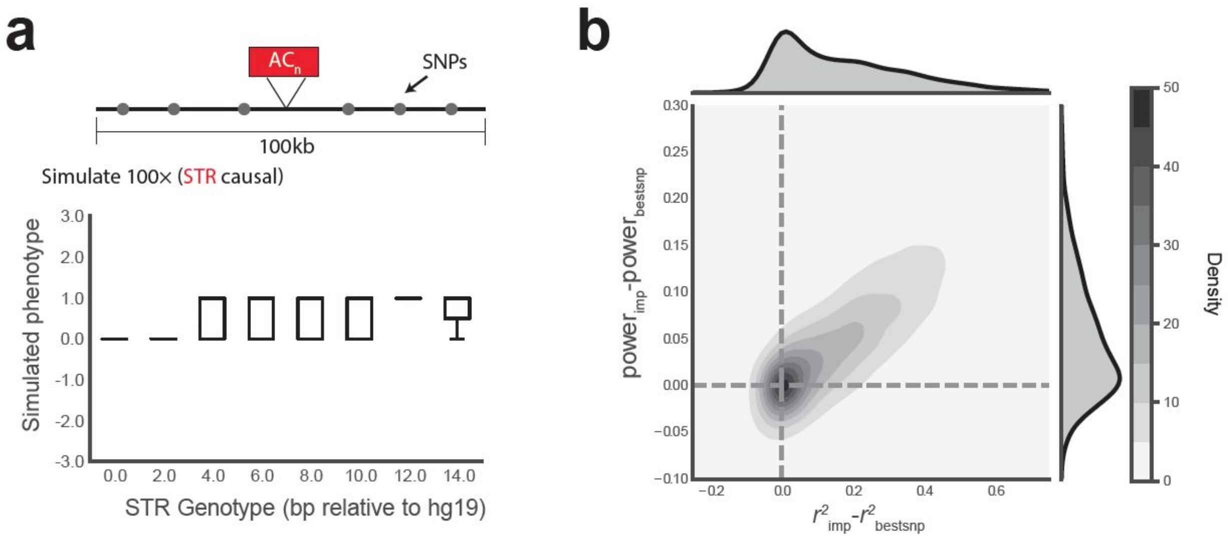

STR imputation improves power to detect STR associations - case control phenotype. a. Example simulated case control phenotype. Simulation is based on observed SSC STR genotypes. Boxes span from the 25th percentile (Q1) to the 75th percentile (Q3). Whiskers extend to Q1-1.5*IQR (bottom) and Q3+1.5*IQR, where IQR gives the interquartile range (Q3-Q1). For case/control simulations all phenotype values are either 0 or 1. **b. The gain in power using imputed genotypes is linearly related to the gain in r^2 compared to the best tag SNP**. Gray contours give the bivariate kernel density estimate. Top and right gray area gives the distribution of points along the x- and y-axes, respectively. Power was calculated based on the number of simulations out of 100 with nominal p-value < 0.05.

Supplementary Figure 9

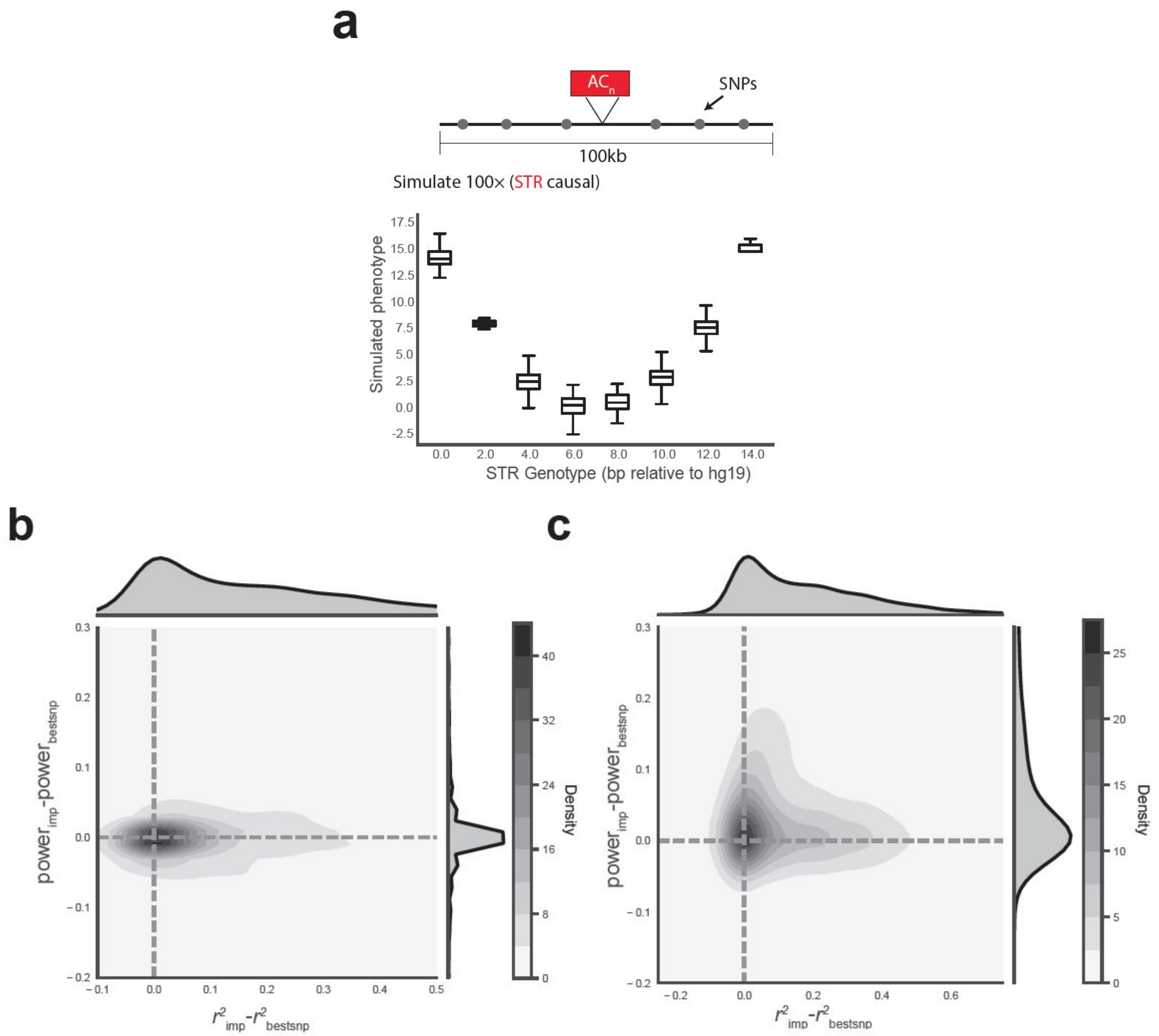

STR imputation improves power to detect STR associations - non-additive phenotype model. a. Example simulated non-additive phenotype. Simulation is based on observed SSC STR genotypes and uses a quadratic model as described in **Online Methods**. Black horizontal lines in the center of each box give median values. Boxes span from the 25th percentile (Q1) to the 75th percentile (Q3). Whiskers extend to Q1-1.5*IQR (bottom) and Q3+1.5*IQR, where IQR gives the interquartile range (Q3-Q1). **b. Gain in power using imputed genotypes compared to the best tag SNP**. STR association tests were conducted by regressing the imputed STR repeat dosage vs. phenotype. **c. Gain in power using per-allele STR association tests compared to the best tag SNP.** A separate association test was performed for each STR allele treating the allele as a bi-allelic marker. For the STR, power was determined using the most strongly associated allele. For **b.** and **c.**, gray contours give the bivariate kernel density estimate. Top and right gray area gives the distribution of points along the x- and y-axes, respectively. Power was calculated based on the number of simulations out of 100 with nominal p-value < 0.05.

Supplementary Figure 10

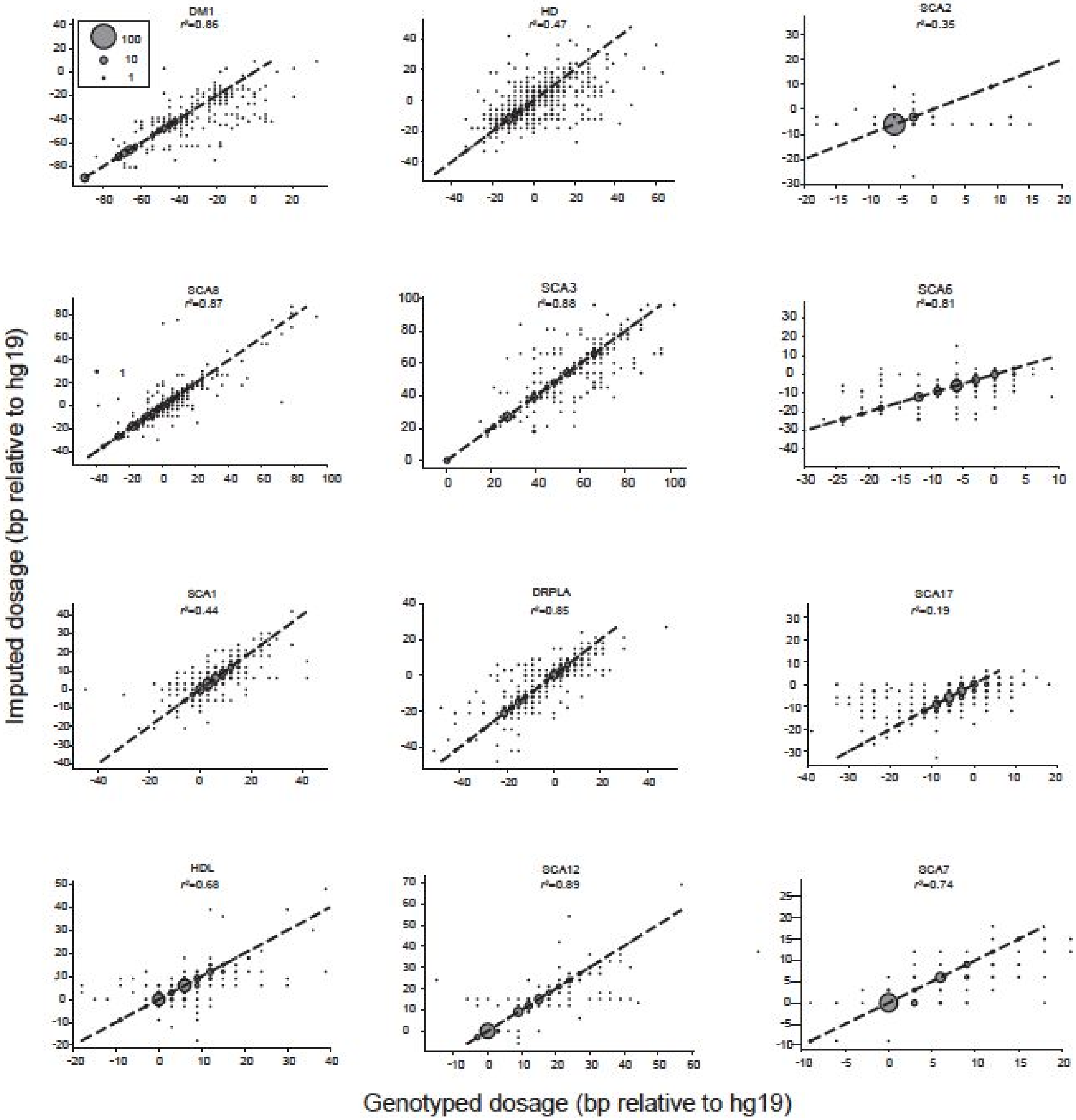

STR imputation performance at known pathogenic STRs. Panels show the genotyped vs. imputed dosage at each locus in the SSC cohort. Dashed lines give the diagonal. Bubble size scales with the number of points represented by each bubble as in **Figure 4**.

Supplementary Table 1

Coordinate (hg19)	Disorder	Locus ID	Motif	Call rate	# Alleles	Tredparse vs. HipSTR concordance
2:176957787	Syndactyly	SD5	GCN	0.01	6.00	
3:128891420	Myotonic dystrophy 2	DM2	CAGG	0.00	-	
3:138664863	Blepharophimosis, epicanthus inversus, and ptosis	BPES	NGC	0.00	-	
3:63898362	Spinocerebellar ataxia 7	SCA7	CAG	0.43	14.00	99.60%
4:3076604	Huntington disease	HD	CAG	0.99	28.00	
4:41747989	Central hypoventilation syndrome	CCHS	NGC	0.00	-	
5:146258292	Spinocerebellar ataxia 12	SCA12	CAG	0.65	21.00	99.90%
6:16327867	Spinocerebellar ataxia 1	SCA1	CAG	0.89	27.00	
6:170870996	Spinocerebellar ataxia 17	SCA17	CAG	0.85	34.00	
6:45390488	Cleidocranial dysplasia	CCD	GCN	0.13	9.00	
7:27239544	Hand-foot-uterus syndrome	HFG	GCN	0.01	5.00	
9:27573527	Amyotrophic lateral sclerosis	ALS	GGCCCC	0.00	-	
9:71652203	Friedreich ataxia	FRDA	GAA	0.00	-	
12:112036755	Spinocerebellar ataxia 2	SCA2	CAG	0.99	20.00	99.40%
12:7045892	Dentatorubral-pallidoluysian atrophy	DRPLA	CAG	0.87	21.00	99.80%
13:100637703	Holoprosencephaly-5	HPE5	GCN	0.01	7.00	
13:70713516	Spinocerebellar ataxia 8	SCA8	CTG/CAG	0.95	36.00	
14:23790682	Oculopharyngeal muscular dystrophy	OPMD	GCN	0.00	-	
14:92537355	Spinocerebellar ataxia 3	SCA3	CAG	0.94	26.00	98.40%
16:87637894	Huntington disease-like 2	HDL	CTG	0.74	20.00	99.70%
19:13318673	Spinocerebellar ataxia 6	SCA6	CAG	0.90	12.00	
19:46273463	Myotonic dystrophy 1	DM1	CTG	1.00	33.00	99.30%
20:2633380	Spinocerebellar ataxia 36	SCA36	GGCCTG	0.00	-	
21:45196325	Unverricht-Lundborg Disease	ULD	CGCGGG GCGGGG	0.00	-	
22:46191235	Spinocerebellar ataxia 10	SCA10	ATTCT	0.00	-	

Set of known pathogenic STRs genotyped using Tredparse. Concordance was estimated for the STRs genotyped using HipSTR. # Alleles gives the number of alleles occurring at least once in the Tredparse calls for SSC.

Supplementary Table 2

Position	ID	Length r^2	Concordance	Edge, *et al.* Concordance	# Alleles	Motif Length
5:149455884	CSF1PO	0.39	63%	60%	10	4
13:82722160	D13S317	0.75	69%	61%	10	4
18:60948895	D18S51	0.60	51%	32%	18	4
19:30417140	D19S433	0.61	70%	NA	15	4
3:45582231	D3S1358	0.66	67%	59%	8	4
5:123111245	D5S818	0.53	70%	60%	9	4
7:83789542	D7S820	0.71	70%	63%	8	4
8:125907107	D8S1179	0.78	69%	59%	10	4
4:155508888	FGA	0.60	48%	41%	17	4
15:97374244	PentaE	0.93	77%	NA	11	5
11:2192318	TH01	0.93	94%	83%	7	4
2:1493425	TPOX	0.87	90%	85%	7	4

Imputation performance at CODIS markers. Values were computed using leave-one-out analysis in the SSC cohort as described in the main text.

Supplementary Table 3

	Platform	Number of samples	Mean conc.	Mean length r^2
**1000 Genomes - EUR**	WGS	49	97.0%	0.91
**1000 Genomes - EUR**	Affy 6.0	8	96.5%	0.86
**1000 Genomes - EUR**	Omni 2.5	50	96.7%	0.90
**1000 Genomes - EAS**	WGS	45	93.8%	0.79
**1000 Genomes - EAS**	Affy 6.0	18	92.4%	0.70
**1000 Genomes - EAS**	Omni 2.5	48	93.4%	0.77
**1000 Genomes - AFR**	WGS	46	90.6%	0.71
**1000 Genomes - AFR**	Affy 6.0	50	87.7%	0.60
**1000 Genomes - AFR**	Omni 2.5	0	-	-

Comparison of imputation performance in 1000 Genomes samples across genotyping platforms. Mean concordance and length r^2 for the different datasets were found by comparing the imputed genotypes against the real genotypes called on high-coverage WGS samples using HipSTR.

Supplementary Table 4

Gene	STR (hg19)	CAVIAR score	HipSTR eSTR *p*-value	Imputed eSTR *p*-value	Best tag SNP	SNP-STR length r^2	Best tag SNP *p*-value
DSCR3	21:38733174	0.35	$2.05x10^{-4}$	$5.0x10^{-5}$	rs9976222	0.46	$3.5x10^{-3}$
CSTB	21:45196326	0.15	$1.36x10^{-12}$	$5.9x10^{-13}$	rs35285321	0.58	$3.2x10^{-9}$
C21orf62	21:34133199	0.094	$2.87x10^{-2}$	$2.9x10^{-1}$	rs9967977	0.77	0.50

Putative causal eSTRs. CAVIAR score gives the posterior probability of causality of the STR. Best tag SNP gives the SNP within 50kb of the STR with highest length r^2.

Supplementary Table 5

Locus	Disease[a]	HipSTR length r^2	HipSTR concordance	HipSTR # alleles	Tredparse length r^2	Tredparse concordance	Tredparse # alleles
3:63898362	SCA7	0.79	92.6%	10	0.80	91.9%	14
5:146258292	SCA12	0.90	94.9%	14	0.90	94.9%	21
12:112036755	SCA2	0.37	95.7%	13	0.48	96.2%	20
12:7045892	DRPLA	0.85	81.6%	19	0.85	81.2%	21
14:92537355	SCA3	0.86	87.1%	20	0.88	86.4%	26
16:87637894	HDL	0.65	88.5%	15	0.70	88.3%	20
19:46273463	DM1	0.88	85.4%	25	0.86	86.9%	33

Comparison of imputation performance using leave-one-out analysis on known pathogenic STRs called using both HipSTR and Tredparse. Tredparse metrics were computed as described in the main text and **Online Methods.** For comparison, HipSTR metrics were re-computed by imputing each STR separately considering all SNPs within a 50kb region surrounding the STR. This was found to give slightly better imputation results compared to imputing all genome-wide STRs simultaneously as is done in the main text. [a]SCA=spinocerebellar ataxia; DRPLA=Dentatorubral-pallidoluysian Atrophy; DM1=Myotonic Dystrophy Type 1; HDL=Huntington's Disease-Like 2.

Supplementary References

1. Gymrek, M., Willems, T., Reich, D. & Erlich, Y. Interpreting short tandem repeat variations in humans using mutational constraint. *Nat. Genet.* **49,** 1495–1501 (2017).
