# Supplementary material for: A reference haplotype panel for genome-wide imputation of short tandem repeats
Source: Nat Commun. 2018 Oct 23;9:4397. doi: 10.1038/s41467-018-06694-0 (PMC6199332; doi:10.1038/s41467-018-06694-0)
Supplement: Supplementary file 3 — Description of Additional Supplementary Files [file 41467_2018_6694_MOESM3_ESM.pdf]

Description of Additional Supplementary Files

File Name: Supplementary Data 1
Description: Per-STR imputation statistics. Imputation concordance and length r^2 for each STR are provided as computed based on (1) leave-one-out analysis in the Simons Simplex Collection Phase 1 data, (2) imputation using whole genome sequencing data from the 1000 Genomes Project and (3) imputation using Omni and Affymetrix SNP data from 1000 Genomes Project samples. For 1000 Genomes Project samples, imputation metrics are listed separately for each population group.

File Name: Supplementary Data 2
Description: Per-allele imputation statistics. Allelic r^2 and allele frequencies are given for each allele at each STR analyzed based on leave-one-out analysis in the Simons Simplex Collection Phase 1 data.
